# Systematic decoding the functional role of human endogenous retrovirus-derived RNAs in medulloblastoma

**DOI:** 10.1093/noajnl/vdag109

**Published:** 2026-04-29

**Authors:** Jiaming Zhao, Wei Yang, Tingting Wang, Jianqi She, Liming Xiao, Changyu Tao, Jian Chen, Ming Ge, Ence Yang

**Affiliations:** Department of Medical Bioinformatics, School of Basic Medical Sciences, Peking University Health Science Center, Beijing, China; Department of Neurosurgery, Beijing Children’s Hospital, Capital Medical University, National Center for Children’s Health, Beijing, China; Beijing Institute for Brain Research, Chinese Academy of Medical Sciences & Peking Union Medical College, Beijing, China; Department of Medical Bioinformatics, School of Basic Medical Sciences, Peking University Health Science Center, Beijing, China; Department of Medical Bioinformatics, School of Basic Medical Sciences, Peking University Health Science Center, Beijing, China; Department of Human Anatomy, Histology and Embryology, School of Basic Medical Sciences, Peking University Health Science Center, Beijing, China; Beijing Institute for Brain Research, Chinese Academy of Medical Sciences & Peking Union Medical College, Beijing, China; Chinese Institute for Brain Research, Beijing, Beijing, China; Changping Laboratory, Beijing, China; Department of Neurosurgery, Beijing Children’s Hospital, Capital Medical University, National Center for Children’s Health, Beijing, China; Department of Medical Bioinformatics, School of Basic Medical Sciences, Peking University Health Science Center, Beijing, China; Department of Microbiology & Infectious Disease Center, School of Basic Medical Sciences, Peking University Health Science Center, Beijing, China

**Keywords:** biomarker, hervRNA, human endogenous retrovirus (HERV), medulloblastoma, non-coding RNA

## Abstract

**Background:**

Medulloblastoma (MB) is the most common malignant pediatric brain tumor, but its pathogenesis remains poorly understood. Although human endogenous retrovirus-derived RNAs (hervRNAs) are implicated in tumorigenesis across various cancers, their functional role in the pathogenesis of MB has not been discovered.

**Methods:**

We collected total RNA samples from 63 pediatric MB patients for RNA sequencing and identified the expression of hervRNAs in a locus-specific manner. The functional role of hervRNAs was analyzed through independent expression analysis, gene set enrichment analysis, weighted correlation network analysis, and further validated by siRNA-knockdown experiment in D283 cells.

**Results:**

We systematically identified 39,613 MB-expressed hervRNAs and found that their expression profile could distinguish the four consensus molecular subgroups. Specifically, 145 subgroup-specific hervRNAs were identified to be significantly associated with tumor development. Functional annotation revealed that these hervRNAs were enriched in known MB subgroup-related pathways but also implied novel pathways related to regulated necrosis, negative regulation of class C GPCR, extracellular matrix remodeling, as well as DNA methylation. Notably, the expression of Group3-specific hervRNAs showed a significant negative correlation with promoter DNA methylation levels. Experimental validation confirmed that Group3-specific *hervRNA_G38830* and *hervRNA_G66017* inhibit cell proliferation and promote apoptosis. Finally, a semi-quantitative model based on 70 hervRNA clusters achieved molecular classification with accuracy above 94.8%.

**Conclusion:**

Our work provided insights into MB tumorigenesis in the layer of hervRNA, uncovering potential targets for therapeutic intervention and novel biomarkers for molecular classification.

Key Points

- The expression of hervRNAs distinguished four consensus molecular subgroups in MB.
- Subgroup-specific hervRNAs were associated with tumor development and overall survival.
- hervRNAs inhibited cellular proliferation and promoted apoptosis in Group 3 MB.

Importance of the Study

Medulloblastoma (MB) is the most common malignant pediatric brain tumor arising in the embryonal cerebellum. Although recent studies have revealed its molecular classification and cellular origins, the complexity and ambiguity of MB pathogenesis still make treatment challenging. In this study, we conducted a comprehensive genome-wide transcriptional profiling of locus-specific human endogenous retrovirus-derived RNAs (hervRNAs) in MB, and identified functional hervRNA targets validated by cell experiment. Our study established foundational understanding of hervRNAs expression pattern in MB and uncovered a new layer of complexity in the molecular mechanism of MB tumorigenesis, which provides potential molecular targets for less toxic and more targeted therapy. Furthermore, we demonstrated hervRNAs can distinguish molecular subgroups with high accuracy. Integrated with protein-coding gene expression and DNA methylation profiles, hervRNAs have the potential to complement traditional methods and achieve a more accurate molecular classification with lower costs in the future.

Medulloblastoma (MB) is one of the most common pediatric central nervous system malignant tumors, accounting for 69.1% of all embryonal tumors in children aged 0-19 years, which makes it an essential cause of child mortality.^1^^,^^2^ Based on multi-omics profiling, heterogeneous MB tumors have been divided into four distinct consensus molecular subgroups: WNT, Sonic Hedgehog (SHH), Group 3, and Group 4.^3^ WNT subgroup tumors are typically driven by mutations in WNT signaling pathway genes,^4^ and the prognosis for patients <16 years of age is excellent as >95% survive beyond 5 years, leaving adults with a less favorable outcome.^5^^,^^6^ Mutations of genes in the SHH signaling pathway lead to SHH MB in the upper rhombic lip granule cell lineage.^7^ Adults with SHH MB are excellent candidates for molecularly targeted therapy with SMO inhibitors, while younger patients treated with SMO inhibitors have severe growth plate complications.^8^ Group 3 (the most aggressive subgroup) and Group 4 (the most prevalent subgroup) tumors share unified beginnings in the rhombic lip in the early stages of human development.^9^ Group 3 tumors are characterized by high-level MYC amplification, along with other notable driver events such as amplifications of MYCN (5% of patients) and OTX2 (3%). In contrast, Group 4 tumors lack predominant somatic gene-level mutations, with no single gene found to be mutated in >10% of cases.^10^ The absence of specific molecular targets in Group 3 and Group 4 subgroups has left treatment largely reliant on conventional strategies, which are associated with significant toxicity and adverse effects on neurodevelopment.^11^ Overall, current therapeutic strategies and patient prognosis of MB remain far from satisfactory, highlighting the urgent need to uncover novel molecular mechanisms involved in MB tumorigenesis and progression.

With the advancement of MB researches on protein-coding genes, long non-coding RNAs (lncRNAs) are recently emerging as another key player in the pathogenesis of MB.^12^^,^^13^ For example, targeted knockdown of the most studied lnc-IRX3-80 and lnc-LRRC47-78 has been shown to elevate apoptosis rates and decrease cell proliferation in MB cells.^14^^,^^15^ In our previous work, we found human endogenous retrovirus-derived RNAs (hervRNAs), a unique subset of lncRNA, were abundantly expressed in the cerebellum and might contribute to the physiology and pathology of cerebellum.^16^ Human endogenous retroviruses (HERVs), members of the long terminal repeat (LTR) retrotransposon repetitive element class, make up an estimated 8% of the human genome.^17^ Although most HERV elements have lost their transcriptional capability due to epigenetic silencing by the host, accumulating evidence suggests that hervRNAs have become “domesticated’” to perform function roles under physiological conditions, such as maintaining pluripotency and regulating early embryogenesis.^18–20^ Beyond their physiological roles, abnormal activation of hervRNAs has been reported in various cancers, frequently driven by epigenetic dysregulation.^21–23^ Within tumor contexts, hervRNAs could contribute to tumor development by encoding novel proteins, regulating gene expression, and activating oncogenic signaling pathways.^24^^,^^25^ Consistent with the abundant expression of hervRNAs in the cerebellum, we noted that hervRNAs also exhibit a temporally dynamic pattern of expression during cerebellar development,^26^ which aligns with the fact that medulloblastoma originates from developing hindbrain. Therefore, we investigated whether hervRNAs could contribute to a cryptic layer of the complex pathogenesis in MB.

To investigate the precise expression patterns and functional contributions of hervRNAs to MB development, we conducted a comprehensive genome-wide transcriptional profiling of locus-specific hervRNAs in MB using our previously established pipeline, Scan Endogenous Retrovirus Expression (SERVE),^16^ and compared their expression pattern with those of normal cerebellum. Further, we systematically identified functional hervRNA candidates and revealed their associated pathways in MB pathogenesis. After conducting methylation analysis and survival analysis, we focused on Group3-specific *hervRNA_G38830* and *hervRNA_G66017*, and verified their function in inhibiting tumor cell proliferation and promoting apoptosis through siRNA knockdown experiment. Finally, we evaluated the potential of hervRNAs to serve as molecular classifiers for MB classification. Collectively, our studies increase the understanding of the expression patterns and functional roles of hervRNAs in MB, providing a novel insight into the tumorigenesis and progression of MB.

## Methods

### Patient Samples

Tumor samples from 63 medulloblastoma patients were collected from Beijing Children’s Hospital. All patient materials were collected after receiving informed consent and under approval by the Medical Ethics Committee of Beijing Children’s Hospital, Capital Medical University ([2021]-E-232-Y).

### Preparation of Library and Sequencings

Total RNA was extracted from tumor tissues using the RNAprep Pure Tissue Kit (DP431, TIANGEN), and ribosomal RNA was removed from total RNA. Short-read RNA sequencing and library preparations for tumors were performed according to the manufacturer’s instructions (Illumina, San Diego, CA, USA). The sequencing was performed in the Illumina NovaSeq platform, generating a ∼60M paired-end reads per sample with a read length of 150 bp.

### hervRNA Identification and Annotation

A genome-guided de novo assembly strategy, SERVE, was conducted to detect locus-specific hervRNA expression as described previously.^16^ To identify independently expressed hervRNAs, Pearson correlation coefficients of TPM between hervRNA and all ENCODE-annotated ENSG genes within 1 Mbp distance of the hervRNA were calculated. A driver gene was defined as a gene with both higher expressions than its hervRNA pair based on a paired Wilcoxon test, and a high correlation (*r *> 0.4) with its hervRNA pair. Independently expressed hervRNA was defined as those without driver genes.

### Functional Annotation of hervRNA

Differential expression analysis was conducted to identify subgroup-specific hervRNAs using DESeq2 package (fold change >2, adjusted *P* value <.05). Pearson correlation coefficients between each subgroup-specific hervRNA and all protein-coding genes were calculated within the specific subgroup samples. The list of protein-coding genes ranked by Pearson correlation coefficient was the subjected to Gene Set Enrichment Analysis (GSEA) by the clusterProfiler package.

A hervRNA-mRNA weighted correlation network was constructed with independently expressed hervRNAs and expressed protein-coding genes in MB using a weighted correlation network analysis (WGCNA) package. Trait-related modules were defined by a strong correlation between module eigengene and molecular subgroups (*r *> 0.4). Within each module, hervRNAs and protein-coding genes were filtered as hub genes based on the following criteria: Module Membership (MM) value >0.8, Gene Significance (GS) >0.2, MM *P* value <0.05, and GS *P* value <0.05. For each differentially expressed hervRNA (DEH) module, Reactome pathway enrichment analysis was conducted on the associated protein-coding genes using the clusterProfiler package.

For evolutionary conservation, primate conservation scores (phastCons30way) of hervRNA promoter regions (defined as TSS −2000 to +500 bp) related to LTR were calculated and compared against non-transcribed whole-genome background LTRs. Motif enrichment and conservation analyses were subsequently performed, defining highly conserved motifs using a strict threshold of phastCons > 0.5. To evaluate the protein-coding potential, intact open reading frames (ORFs) within the hervRNA transcripts were predicted using TransDecoder. To assess RNA structural stability, the global secondary structures of the hervRNA transcripts were computationally folded using RNAfold. Stable structured hervRNAs were defined by normalized minimum free energy (MFE) ≤ −0.25 and a normalized ensemble diversity ≤ 0.15.

### NMF Analysis

Consensus non-negative matrix factorization (NMF) analysis was performed to obtain the subgroup of medulloblastoma samples. The raw read count matrix was normalized through DESeq2 vst function. Protein-coding genes with top 10% standard deviation across samples or independent hervRNA were selected for further analysis. NMF algorithm was conducted using the nmf function from the NMF R package (rank = 4, nrun = 200). Each cluster was aligned to different molecular subgroup of MB according to the KEGG enrichment of its differentially expressed genes.

### DNA Methylation Analysis

We analyzed whole genome bisulfite sequencing (WGBS) data from EGAD00001001630 with corresponding RNA-seq data from EGAD00001001620. Bisulfite-converted reads were trimmed using fastp (v0.23.4) and mapped to the human genome (GRCh38) using Bismark (v0.24.2). After deduplication, methylation was quantified through methylKit (v1.26.0), and only CpG sites with at least five reads and coverage across all samples were retained. Promoter regions of hervRNAs were defined as the TSS ± 1,000 bp. Differential methylation of hervRNA promoter was calculated using calculateDiffMeth function (difference = 25, *q* value = 0.01).

### Survival Analysis

Survival analysis was conducted using a publicly available bulk RNA-seq dataset (EGAD00001004435). The patient cohort was stratified into two groups based on the median TPM expression. Overall survival for each class of Group 3 MB patients was analyzed using the Kaplan-Meier method. *P*value were determined using the log-rank test. The analysis was performed using the R package survival (v.3.5-7), and visualized by survminer packages (0.4.9).

### Cell Culture and RNA Isolation

MB cell line D283 (Procell, CL-0271, China) was cultured in MEM (containing NEAA) medium (Procell, PM150410, China) with 10% FBS and 1% penicillin/streptomycin at 37°C in an atmosphere of 5% CO_2_. RNA was isolated from cell pellets using the FastPure Cell/Tissue Total RNA Isolation Kit V2 (Vazyme, RC112-01).

### Transcript Validation

The isolated total RNA was subsequently reverse transcribed into cDNA by using a HiScript III 1st Strand cDNA Synthesis Kit (Vazyme, R312-01). We designed primers at both ends of the hervRNA (Supplementary Table S1) and amplified the cDNAs with 2× Rapid Taq Master Mix (Vazyme, P222). The final PCR products were analyzed by 1% agarose gel electrophoresis and sequenced by Sanger sequencing.

### hervRNA Knockdown

At 40% to 50% confluency, cells were transfected with a 10-nM concentration of *hervRNA_G38830*-target siRNA or *hervRNA_G66017*-target siRNA (Supplementary Table S2) or non-targeting control siRNA that had been formulated with CALNP RNAi in vitro transfection reagent (D-Nano, DN001-05). Three siRNA were designed for each hervRNA, and the siRNA with the highest knockdown efficiency was used for downstream experiments.

### Cell Apoptosis Assays

Cell apoptosis was analyzed by flow cytometry with a FITC-Annexin V Apoptosis Detection Kit (Yeasen Biotech, 40302ES20). After 72 h of siRNA transfection, cells were harvested and double-stained by FITC-Annexin V and propidium iodide, followed by flow cytometry (Calibur2).

### Cell Proliferation Assays

Cell proliferation was assessed using EdU assay (Beyotime, C0071S). 72 h of post-transfection, cells were treated with 10 µM EdU for 3 h, washed with PBS, fixed in 4% PFA (15 min), permeabilized with 0.3% Triton X-100 (10 min), and washed with 3% BSA/PBS. After click-reaction (30 min) and final washes, proliferation was quantified by flow cytometry (Fortessa).

In Cell Counting Kit-8 (CCK-8) assay, the non-targeting control cells and hervRNA-knockdown cells were seeded into 96-well plates. 10 µL of Cell Counting Kit-8 (Beyotime, C0038) solution were added to each 96-well at 24, 48, 72, and 96 h. After 2 h of incubation, we recorded optical density at 450 nm.

### MB Semi-Quantitative Classification by hervRNA

The expression of hervRNAs was transformed to binary variable based on the threshold of TPM = 1. hervRNAs meeting the following conditions were retained as following classifiers: 1) The expression was significantly associated with subgroup (Fisher’s exact test, adjusted *P*value < 0.01). 2) Subgroup-specific expression (mean TPM > 5 in target subgroup and < 5 in others). 3) The log_2_ fold change of the mean TPM > 2 between the target subgroup and other subgroups. 4) Classification specificity > 0.8. The identified hervRNA classifiers were subsequently grouped into 70 hervRNA clusters based on a maximum distance of 200 kbp between elements. Expression of any hervRNA within a cluster indicated overall cluster expression. The subgroup with the highest cluster expression rate was defined as final subgroup classification.

### Statistical Analysis

All statistical analyses were performed using RStudio (https://www.rstudio.com/ and R v.4.3.2, R Development Core Team, 2023). We use the ‘cor’ function to calculate the Pearson correlation coefficient. Statistical significance was determined using two-tailed Student’s *t*-tests for normal distribution data and Wilcoxon test for data without distribution. Enrichment analysis was performed with a two-tailed Fisher’s exact test, and all *P* value were corrected using the BH method. Data are shown as mean ± S.E.M. Values of *P *< .05 denoted a statistically significant difference.

## Results

### Transcriptional Landscape of Locus-Specific hervRNAs in MB

To profile the transcriptome of human endogenous retrovirus-derived RNAs (hervRNAs) in medulloblastoma (MB), we collected 63 clinical samples of pediatric MB patients from Beijing Children’s Hospital (Supplementary Table S1). After conducting rRNA-depleted strand-specific RNA sequencing, an average of 59.1 million clean paired-end reads per sample (17.6 Gbp) was generated. Through hervRNA identification pipeline, SERVE,^16^ a total of 39,613 locus-specific hervRNAs were identified across the dataset (Supplementary Table S2).

**Figure 1.**
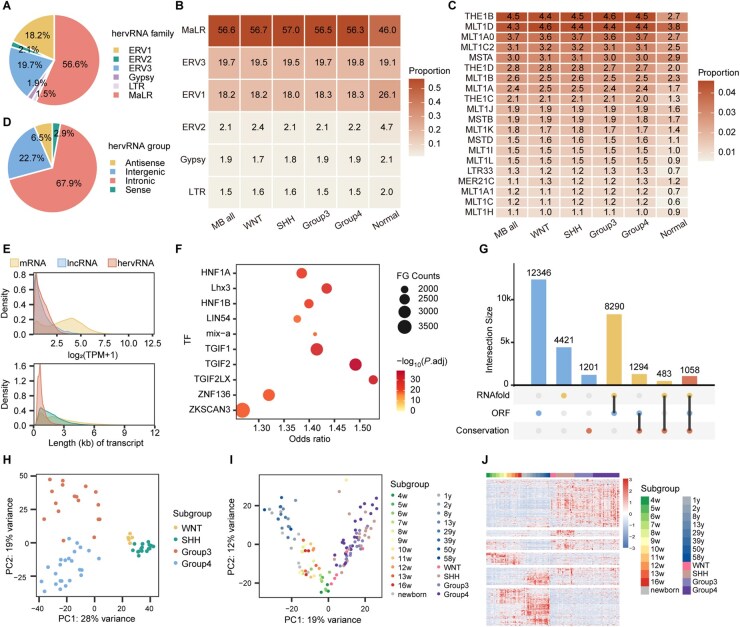
The transcriptional landscape of locus-specific hervRNAs in medulloblastoma. (A) Pie chart showing the distribution of hervRNA families in the overall MB cohort, annotated using the Dfam database. (B) Heatmap displaying the mean proportion of hervRNA families across the full MB cohort, four molecular subgroups (WNT, SHH, Group 3, Group 4), and normal cerebellar tissues. (C) Heatmap of mean proportions of the most abundant TE subfamilies across the same sample groups as in (B). Rows represent individual subfamilies; columns correspond to sample groups. (D) Pie chart showing the genomic distribution of expressed hervRNAs relative to protein-coding genes, categorized as intronic, intergenic, antisense, or sense. (E) Density plots of hervRNA expression characteristics compared with protein-coding mRNAs and lncRNAs. Upper panel: distribution of log_2_(TPM + 1) expression levels; lower panel: distribution of transcript lengths in kilobases (kb). (F) Dot plot of transcription factor (TF) motif enrichment analysis in expressed hervRNAs’ LTR region versus genomic LTR background without hervRNA expression. X-axis shows odds ratio; dot size represents the number of hervRNAs containing the motif; dot color indicates statistical significance. (G) UpSet plot showing the overlap among three features of expressed hervRNAs: conserved LTR motif, presence of intact open reading frames (ORFs), and predicted stable secondary structures by RNAfold. Horizontal bars at the bottom indicate the size of each individual set; vertical bars show the intersection sizes, with numbers indicating the count of hervRNAs in each combination. (H) Principal component analysis (PCA) of hervRNA expression profiles in MB samples. Each point represents one sample and is colored by molecular subgroup. (I) PCA of the 1,365 hervRNAs shared between MB and a normal human cerebellar development dataset (E-MTAB-6814). Each point represents one sample and is colored and shaped by both molecular subgroup and developmental/postnatal age group. w, week. y, year. (J) Heatmap of normalized expression of the 1,365 shared hervRNAs across MB samples. Top annotation bars indicate sample age group and molecular subgroup.

With annotation using the Dfam database,^27^ the most abundant hervRNA family in MB was MaLR (56.60%), followed by ERV3 (19.73%) and ERV1 (18.17%) (Figure 1A). When comparing across the four molecular subgroups and normal cerebellar tissues, we found high consistency in the family composition among the four subgroups, with MaLR showing significantly higher activation than in normal samples (Figure 1B). At the TE subfamily level, THE1B, MLT1D, MLTA0, and MLT1C2 were the most prevalent subfamilies in both the overall MB cohort and the individual subgroups, whereas normal cerebellar tissues exhibited a distinctly different distribution pattern (Figure 1C). Based on genomic location related to protein-coding genes,^28^ expressed hervRNAs were categorized into four groups: 26,895 intronic hervRNAs, 8977 intergenic hervRNAs, 2588 antisense hervRNAs, and 1153 sense hervRNAs (Figure 1D). Regarding expression levels, the average transcripts per million (TPM) of hervRNAs (1.36 ± 0.72, mean ± SE) was slightly lower than that of lncRNAs (3.93 ± 1.08) and significantly lower than protein-coding genes (24.30 ± 0.72) (Figure 1E). Taken together, these findings demonstrated that hervRNAs expressed in MB are predominantly intronic, characterized by low expression levels and shorter transcript lengths ­compared to other lncRNAs and mRNAs.

To further evaluate the functional potential of hervRNAs, we systematically annotated them based on evolutionary constraint, coding capacity, and structural stability (Supplementary Table S3). Evolutionary analysis revealed that long terminal repeat (LTR) region of hervRNA exhibited significantly higher conservation scores across primates compared to genomic background LTRs (0.1118 vs. 0.0935, *P *< .001) (Supplementary Figure S1A-D). Furthermore, motif enrichment analysis identified 4,517 hervRNAs harboring conserved transcription factor binding motifs, especially enriched for TGIF family and HNF1A/B (Figure 1F). Regarding coding and structural potential, 58.03% (22,988) of the expressed hervRNAs maintained intact open reading frames (ORFs) (Supplementary Figure S1E), and 35.98% (14,252) were predicted to fold into highly stable secondary structures (Supplementary Figure S1F-H). Taken together, a total of 29,093 hervRNAs (73.44%) met at least one criterion of potential function annotation (Figure 1G).

### The Expression Profile of hervRNAs in Medulloblastoma Was Associated With Molecular Subgroup and Cerebellar Origin

To determine whether the transcriptional profile of hervRNAs could reflect the heterogeneity of the four consensus molecular subgroups, we first performed the non-negative matrix factorization (NMF) algorithm and consensus clustering to categorizing the 63 MB samples into four molecular subgroups (Supplementary Figure S2A). After principal component analysis (PCA) based on the expression level of 39,613 expressed hervRNAs, the distribution of samples showed a clear separation of four distinct clusters (Figure 1H), suggesting that the expression patterns of hervRNAs are closely associated with the distinct biological characteristics and pathobiology of different MB subgroups.

To investigate whether the hervRNA transcriptome could capture aspects of abnormal cerebellar development. A total of 5,444 hervRNAs were identified across a normal cerebellum development dataset (E-MTAB-6814)^29^ using the same pipeline, with 1,365 overlapping between MB and the normal cerebellum. PCA results of the 1365 shared hervRNAs delineated a trajectory corresponding to developmental stage in the normal cerebellum samples. Remarkably, the MB samples approximated the trajectory of the normal hindbrain at 4-7 pcw (Figure 1J), indicating the similarity of hervRNA expression between medulloblastoma and early hindbrain development. Furthermore, the expression pattern of the 1365 shared hervRNAs demonstrated that some hervRNAs expressed increasingly during development were downregulated in MB, whereas others that typically decreased during development remained highly expressed in MB (, Supplementary Figure S2B and C), suggesting a dysregulation of hervRNAs associated with developmental timing. These findings underscored the role of hervRNAs in reflecting fetal transcriptional characteristics and suggested that hervRNAs might be involved in the tumorigenesis of MB through disruptions in early cerebellar development.

### Potential Function of Subgroup-Specific Independently Expressed hervRNAs

**Figure 2.**
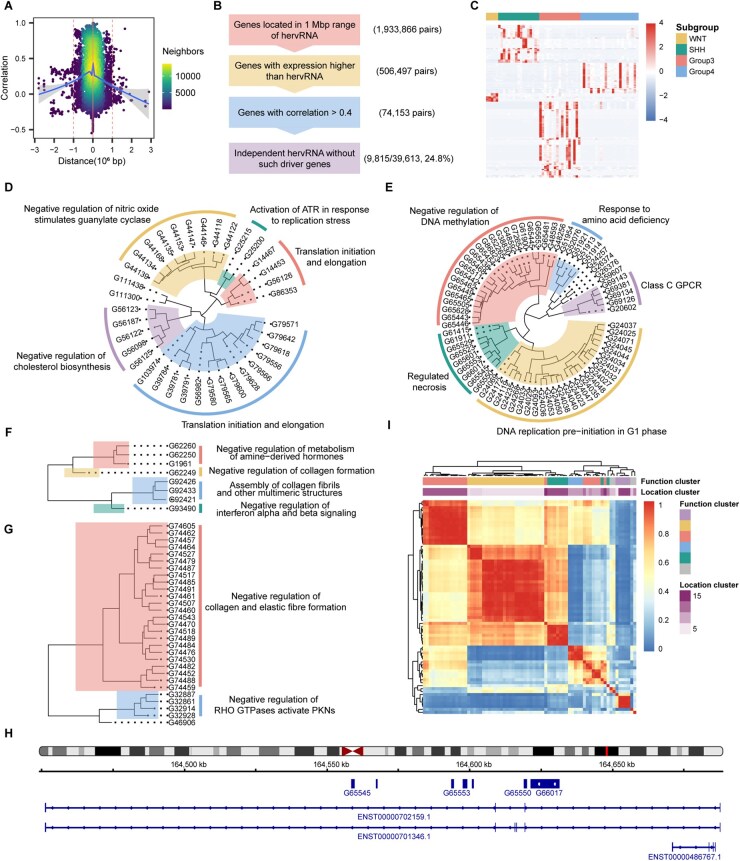
Potential function of subgroup-specific independently expressed hervRNAs. (A) Density scatter plot illustrating the genomic distance and correlation between the expression of hervRNAs and their nearest protein-coding genes. The distribution demonstrates that passively co-transcribed hervRNAs are predominantly located within a ±1.0 Mbp window, where the majority of highly correlated pairs (*r* > 0.4) are captured. (B) Schematic overview of the computational filtering pipeline designed to identify independently expressed hervRNAs. By systematically excluding hervRNAs located within 1.0 Mbp of any annotated gene that exhibits both higher expression and significant co-expression (*r* > 0.4), the pipeline successfully filters out elements passively co-transcribed due to shared chromatin domains, isolating 9,815 independently expressed hervRNAs. (C) Heatmap depicting the expression profiles of subgroup-specific independently expressed hervRNAs (filtered by TPM > 1). Columns represent individual MB patient samples categorized by the four consensus subgroups (WNT, SHH, Group 3, and Group 4), demonstrating the sparse, highly subgroup-specific expression patterns and inter-tumor heterogeneity. (D-G) Hierarchical clustering dendrograms detailing the functional annotations of subgroup-specific hervRNAs for (D) SHH, (E) Group 3, (F) WNT, and (G) Group 4 subgroups. The functional classes were defined based on the Normalized Enrichment Scores (NES) derived from Gene Set Enrichment Analysis (GSEA) of hervRNA-co-expressed protein-coding genes using the Reactome pathway database. Representative enriched pathways for each subgroup are highlighted. (H) Integrative Genomics Viewer (IGV) browser screenshot displaying the genomic coordinates of a representative cluster of Group 3-specific hervRNAs, illustrating their spatially proximal distribution along the genome. (I) Correlation matrix heatmap evaluating the co-expression relationships among Group 3-specific independently expressed hervRNAs. Top annotations denote the functional clusters (corresponding to the biological pathways in panel E) and locational clusters (representing physical genomic proximity).

To identify putative functional Figure 1Gwhile excluding those passively co-transcribed due to shared open chromatin domains or topologically associating domains (TADs), we systematically filtered for independently expressed hervRNAs without co-expression with adjacent genes. We found that nearly half of the expressed hervRNAs (19,668/39,613, 49.65%) were co-expressed (*r *> 0.4) with their nearest protein-coding gene (Figure 2A). Parameter sensitivity analysis established ±1.0 Mbp as the optimal distance threshold for adjacent genes, capturing 99.89% of highly correlated nearest-neighbor pairs (Supplementary Figure S3A). This threshold was further supported by 3D genomic data, aligning with the average MB TAD size of ∼1.5 Mbp (Supplementary Figure S3B). By strictly filtering out hervRNAs correlated with any annotated ENSG gene (both protein-coding and non-coding transcripts) within this ±1.0 Mbp boundary (Figure 2B), we identified 9,815 independently expressed hervRNAs. These hervRNAs exhibited comparable expression to co-expressed hervRNAs (mean TPM 1.28 ± 0.02 vs. 1.39 ± 0.01, *P *= 4.37 × 10^−4^) and significant intergenic enrichment (4,143/9,815 vs. 4,834/29,798 in co-expressed hervRNAs, *P *< 1 × 10^−300^) (Supplementary Figure S3C). This suggested independent hervRNAs maintain stable expression without proximal gene enhancement, indicating their capacity for relatively independent and autonomous transcription.

To further explore functional hervRNAs across MB subgroups, we focused on differentially expressed hervRNAs with TPM > 1, revealing 8 WNT-specific, 36 SHH-specific, 72 Group 3-specific and 29 Group 4-specific hervRNAs (Supplementary Table S4). Their sparse expression reflected individual heterogeneity (Figure 2C). For each subgroup-specific hervRNA, we performed Gene Set Enrichment Analysis (GSEA) of co-expressed protein-coding genes using the Reactome pathway database.^30^ The top five Reactome terms with the highest Normalized Enrichment Score (NES) were identified for each subgroup-specific hervRNA (Supplementary Table S5). Hierarchical clustering based on NES revealed distinct functional classes for each subgroup’s hervRNAs (Supplementary Figure S3D). For example, SHH-specific hervRNAs were linked to translation initiation and elongation, negative regulation of nitric oxide stimulates guanylate cyclase, activation of ATR in response to replication stress, and cholesterol biosynthesis (Figure 2D). Group 3-specific hervRNAs were enriched in pathways involving negative regulation of DNA methylation, regulated necrosis, DNA replication pre-initiation in G1 phase, class C GPCR, and response to amino acid deficiency (Figure 2E). WNT-specific hervRNAs were significantly associated with pathways related to interferon alpha and beta signaling, the assembly of collagen fibrils and other multimeric structures, collagen formation, and metabolism of amine-derived hormones (Figure 2F). Lastly, Group 4-specific hervRNAs were predominantly associated with negative regulation of collagen and elastic fiber formation, negative regulation of assembly of collagen fibrils and other multimeric structures, and negative regulation of RHO GTPases activating PKNs (Figure 2G).

Further analysis of the genomic distribution of subgroup-specific hervRNAs revealed significant genomic clustering (Figure 2H). These spatially proximal hervRNAs exhibited strong co-expression (Figure 2I) and aligned with functional classifications. These findings demonstrated that subgroup-specific hervRNAs are characterized by clustered distribution patterns across the genome.

### Subgroup-Specific Independently Expressed hervRNAs Were Enriched in Tumor-Related WGCNA Modules

**Figure 3.**
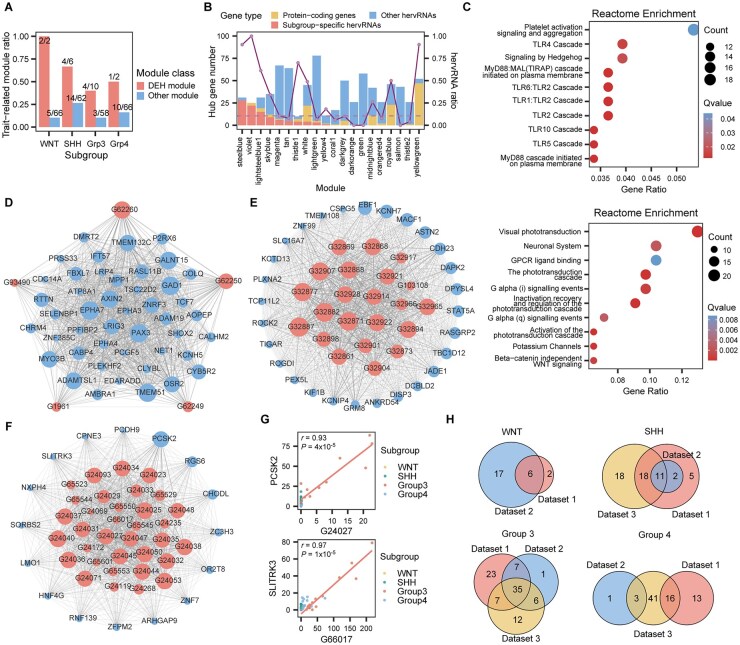
Subgroup-specific independently expressed hervRNAs occupy central regulatory hubs in tumor-associated co-expression networks. (A) Bar chart comparing the proportion of trait-related modules, defined by strong correlation between module eigengene and molecular subgroups (*r* > 0.4), between differentially expressed hervRNA (DEH) modules and other non-DEH modules. (B) Dual-axis plot detailing the hub gene composition within the 20 identified DEH modules. Stacked bars (left y-axis) represent the absolute number of hub genes categorized by gene type (protein-coding genes, other hervRNAs, and subgroup-specific hervRNAs). The connected red dots (right y-axis) indicate the ratio of hervRNAs among all hub genes within each module. The horizontal dashed line denotes the baseline average proportion of hub hervRNAs in non-DEH modules. (C) Bubble plots displaying Reactome pathway enrichment for protein-coding genes within the SHH-associated “green” module (top) and the Group 3-associated “salmon” module (bottom). Bubble size corresponds to the enriched gene count, and color gradient represents the *Q*-value. (D-F) Gene co-expression network diagrams illustrating the topological interactions between central hervRNAs (red nodes) and protein-coding genes (blue nodes) within specific DEH modules: (D) WNT module “tan”, (E) Group 4 module “white”, and (F) Group 3 module “steelblue”. Node size reflects the Module Membership (MM) score, indicating network centrality, while edge width represents the co-expression weight. (G) Scatter plots demonstrating the strong positive linear correlation between specific hub hervRNAs and essential tumor-related genes within the Group 3 “steelblue” module. Expression levels are quantified in transcripts per million (TPM) and colored by MB subgroup. (H) Venn diagrams illustrating the reproducibility of the identified subgroup-specific independently expressed hervRNAs across three independent cohorts: Dataset 1 (in-house discovery cohort), Dataset 2 (EGAD00001001620), and Dataset 3 (EGAD00001004435).

To explore the regulatory potential of subgroup-specific independently expressed hervRNAs, weighted correlation network analysis (WGCNA) of protein-coding genes and 4,121 independent hervRNAs with TPM > 1 identified 68 co-expression modules. Subgroup-specific independently expressed hervRNAs distributed across 20 differentially expressed hervRNA (DEH) modules: 2 WNT modules, 6 SHH modules, 10 Group 3 modules and 2 Group 4 modules. Compared to non-DEH modules, the DEH modules exhibited a significantly higher proportion of trait-related modules (Figure 3A, Supplementary Figure S4). Further investigation into the hub genes revealed that a higher proportion of hub genes were hervRNAs within DEH modules, compared to the average proportion of non-DEH modules (Figure 3B). Specifically, a substantial fraction of subgroup-specific independently expressed hervRNAs (97 out of 145) were identified as module hub genes. These findings highlighted that subgroup-specific independently expressed hervRNAs frequently occupied central hub positions within their respective gene co-expression networks.

To investigate DEH modules’ relevance to tumor biology, we performed Reactome enrichment analysis on the protein-coding genes in each DEH module. SHH DEH module “green” showed significant enrichment in Hedgehog ‘on’ state, which was well-established in the pathogenesis of SHH MB,^7^^,^^31–33^ as well as Toll-like receptor cascade (Figure 3C). In the Group 3 DEH module “salmon,” pathways were related to the photoreceptor transcriptional program, which was reported to be associated with tumorigenesis of Group3 MB^9^^,^^34^ (Figure 3C). Other Group 3 DEH modules also exhibited enrichment in GPCR ligand binding, expression and translocation of olfactory receptors, and ERBB2 regulates cell motility. By annotating tumor-related genes in DEH modules using the COSMIC database,^35^ we found that WNT DEH module “tan” contained 22 oncogenes, such as *AXIN2*, *LRIG3*, *PAX3*, *EPHA7*, *ZNRF3*, etc. (Figure 3D), while Group 4 DEH module “white” encompassed 6 oncogenes, including *ABL2*, *ACVR1*, *EBF1*, *PCM1*, *STAT3*, and *TSC1* (Figure 3E). In Group 3 DEH module “steelblue” (Figure 3F), essential genes like *PCSK2*, which is involved in proteolytic activation of polypeptide hormones and neuropeptide precursors and known to express in neuroendocrine tumors,^36^ exhibited strong correlation with *herv_G24027* within Group 3 subgroup (Figure 3G). Additionally, we also found correlations between *herv_G66017* and *SLITRK3*, a synapse development gene implicated as an oncogene in multiple cancers^37^^,^^38^ (Figure 3G). These results indicated that subgroup-specific independently expressed hervRNAs were mainly distributed in WGCNA modules enriched for tumor-related pathways and oncogenes, indicating their potential regulatory roles in MB pathogenesis.

To verify the robustness of subgroup-specific independent hervRNA expression, we analyzed another two publicly available datasets (EGAD00001001620 and EGAD00001004435). Our validation revealed that 11 SHH-specific hervRNAs (11/36, 30.6%) and 35 Group 3-specific hervRNAs (35/72, 48.6%) were consistently identified across all three datasets, with 31 SHH-hervRNAs and 55 Group 3-hervRNAs validated in at least two datasets (Figure 3H). However, only 19 Group 4-specific hervRNAs were confirmed by two datasets, demonstrating a more robust expression pattern of Group 3-specific hervRNAs compared to other subgroups.

### Differentially High Expression of hervRNAs in Group 3 Was Associated With Hypomethylation on Promoter Regions

**Figure 4.**
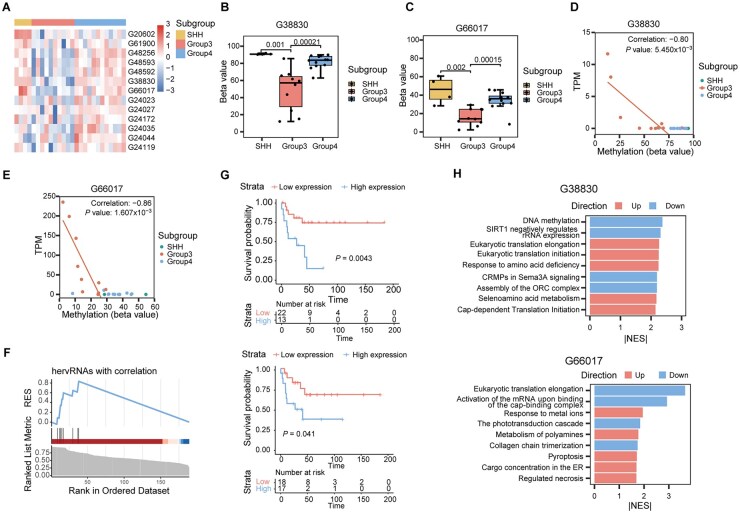
Differentially high expression of hervRNAs in Group 3 was associated with hypomethylation on promoter regions. (A) Heatmap depicting the DNA methylation levels (beta values) within the promoter regions (TSS ± 1,000 bp) of 13 differentially hypomethylated, Group 3-specific hervRNAs across MB subgroups. (B-C) Boxplots detailing the specific promoter methylation levels (beta values) of two key functional candidates, (B) *hervRNA_G38830* and (C) *hervRNA_G66017*, across SHH, Group 3, and Group 4 subgroups. The indicated *P*-values demonstrate significant hypomethylation exclusively within Group 3 MB. (D, E) Scatter plots demonstrating the negative correlation between promoter methylation (beta value, x-axis) and transcript expression level (TPM, y-axis) for (D) *hervRNA_G38830* (*r* = −0.80) and (E) *hervRNA_G66017* (*r* = −0.86). Dots are color-coded by molecular subgroup. (F) Enrichment plot evaluating the topological importance of eight hypomethylated, negatively correlated hervRNAs within the Group 3-specific “steelblue” weighted correlation network analysis (WGCNA) module. Genes are ranked in descending order by their Module Membership (MM) values. The significant accumulation of these hervRNAs at the top of the ranked list (left) indicates their strong connectivity and central regulatory roles within the subgroup-specific network. (G) Kaplan-Meier overall survival curves for Group 3 MB patients stratified by the expression levels of *hervRNA_G38830* (top) and *hervRNA_G66017* (bottom). Patients were divided into high- and low-expression strata based on the mean TPM. *P*-values were calculated using the log-rank test. (H) Bar charts summarizing the functional annotations of *hervRNA_G38830* (top) and *hervRNA_G66017* (bottom). The pathways represent the top Reactome terms enriched among their co-expressed protein-coding genes. The x-axis indicates the absolute Normalized Enrichment Score (|NES|), and bar colors define the direction of the pathway association.

Although hervRNAs are transcriptionally silenced in physiological conditions, they can be reactivated as a result of dysregulation of DNA methylation in cancers,^24^^,^^25^^,^^39^ consistent with the observed negative regulation of DNA methylation in the above pathway analysis for hervRNAs. To investigate the potential regulatory mechanisms of promoter methylation on hervRNAs in MB, we integrated transcriptome and methylation data from 26 individuals (EGAD00001001620 and EGAD00001001630). Focusing on Group 3 subgroup, which exhibited the most robust expression of subgroup-specific hervRNAs, our analysis discovered that 13 out of 72 Group 3-specific hervRNAs showed significantly differential hypomethylation in their promoter regions compared to other subgroups (Figure 4A-C). These differentially methylated hervRNAs also displayed high expression levels specific to Group 3, as verified in the corresponding external RNA-seq datasets (Supplementary Figure S5A). Further investigation revealed nine Group 3-specific independent hervRNAs showing a significant negative correlation between promoter methylation and expression (*r* < −0.6, *P *< .05) among Group 3 samples (Figure 4D and E). Notably, eight of these nine hervRNAs were distributed in the same hervRNA-dominant module “steelblue,” and ranked among the top important genes based on Module Membership values (Figure 4F), indicating their strong connectivity and importance within the network.

### Knockdown of hervRNAs in Group 3 Promoted Tumor Proliferation and Inhibited Apoptosis

According to the expression pattern and methylation status, we focused on the Group 3-specific independently expressed *hervRNA_G38830* and *hervRNA_G66017* (Figure 4B-E). The expression of *hervRNA_G38830* and *hervRNA_G66017* was also cross-validated by two independent clinical cohorts (Supplementary Figure S5A and B). Critically, survival analysis indicated a significant association between overall survival and the expression of *hervRNA_G38830* and *hervRNA_G66017* (*P *= .0043 and *P *= .041, respectively) (Figure 4G). To further annotate their potential functions, Reactome enrichment of protein-coding genes co-expressed with *hervRNA_G38830* revealed positive enrichment in pathways related to amino acid deficiency, and selenoamino acid metabolism, as well as down-regulated pathways including DNA methylation, CRMPs in Sema3A signaling, and assembly of ORC complex (Figure 4H). While *hervRNA_G66017* was implicated in up-regulated pathways such as sequestering heavy metals, polyamine metabolism, pyroptosis, and TP53 acetylation activation, and negative enrichment in translation initiation and elongation, phototransduction cascades, and collagen trimerization (Figure 4H).

**Figure 5.**
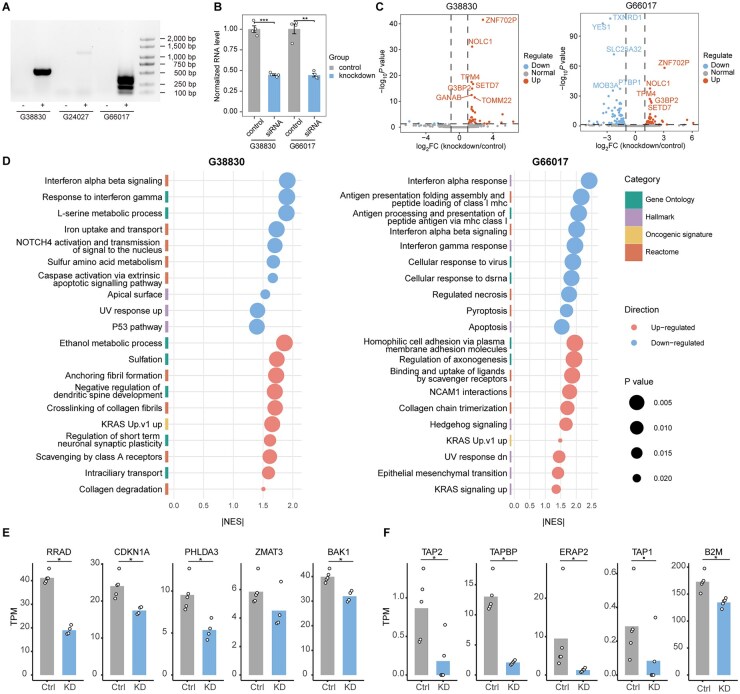
Transcriptomic landscape alterations following the targeted knockdown of Group 3-specific hervRNAs. (A) Representative agarose gel electrophoresis image validating the endogenous transcription of Group 3-specific candidates (hervRNA_G38830, hervRNA_G24027, and hervRNA_G66017) in the D283 medulloblastoma cell line via reverse transcription PCR (RT-PCR). The rightmost lane indicates the DNA marker ladder for size reference. (B) Bar chart evaluating the siRNA-mediated knockdown efficiency of hervRNA_G38830 and hervRNA_G66017 in D283 cells as determined by RT-qPCR. Data are normalized to a non-targeting control siRNA. Error bars represent mean ± SE. (C) Volcano plots illustrating the differentially expressed genes (DEGs) identified by RNA sequencing following the knockdown of hervRNA_G38830 (left) and hervRNA_G66017 (right). The x-axis indicates the log_2_(fold change), and the y-axis represents the -log_10_*P*-value. Significantly up-regulated and down-regulated genes are denoted respectively. (D) Bubble plots summarizing the Gene Set Enrichment Analysis (GSEA) results depicting the global pathway shifts post-knockdown of hervRNA_G38830 (left) and hervRNA_G66017 (right). The x-axis displays the absolute Normalized Enrichment Score (|NES|). Bubble size reflects the *P*-value, while color coding denotes the functional category and the direction of pathway regulation. (E) Bar charts comparing the TPM expression levels of key downstream effector genes of the P53 signaling pathway between the control (Ctrl) and *hervRNA_G38830* knockdown (KD) groups. (F) Bar charts displaying the TPM expression levels of core structural and processing components of the class I MHC antigen-presentation pathway in Ctrl versus *hervRNA_G66017* KD cells.·, *P* < .1. *, *P* < .05. **, *P* < .01. ***, *P* < .001.

To experimentally confirm the functions of Group 3-specific *hervRNA_G38830* and *hervRNA_G66017*, we first verified their transcript sequences using reverse transcription PCR (RT-PCR) (Figure 5A) and Sanger sequencing in D283 cell lines cultured in-house. Subsequent siRNA transfection in D283 cells significantly knocked down both hervRNA transcripts compared to non-targeting siRNA control (Figure 5B). To investigate transcriptome changes of knockdown, RNA sequencing identified 13 differentially expressed genes (13 up-regulated genes) between siRNA_G38830 and siRNA_control, and 77 differentially expressed genes (24 up-regulated genes and 53 down-regulated genes) after *hervRNA_G66017* knockdown (Figure 5C, Supplementary Table S6). There were several genes which were up-regulated after both *hervRNA_G66017* and *hervRNA_G66017* knock-down, including histone methyltransferase *SETD7*, and development-related gene *OGFR*, *NOLC1*, *NeuroD2*, and *FOXP4*. GSEA analysis indicated that *hervRNA_G38830* knockdown up-regulated pathways involved in KRAS signaling, while down-regulating P53 pathway, interferon signaling, and NOTCH4 activation (Figure 5D, Supplementary Figure S6A). Key downstream effectors of P53 pathway, including *CDKN1*, *BAK1*, *PHLDA3*, *ZMAT3*, and *RRAD* were consistently downregulated (Figure 5E). In addition, *hervRNA_G66017* knockdown up-regulated pathways including SHH signaling, epithelial mesenchymal transition (EMT), KRAS signaling, and down-regulated class I MHC antigen-presentation pathway, interferon signaling, and apoptosis (Figure 5D, Supplementary Figure S6B). Core components responsible for peptide transport (*TAP1*, *TAP2*), trimming (*ERAP2*), and complex stabilization (*TAPBP*, *B2M*) were significantly downregulated (Figure 5F).

**Figure 6.**
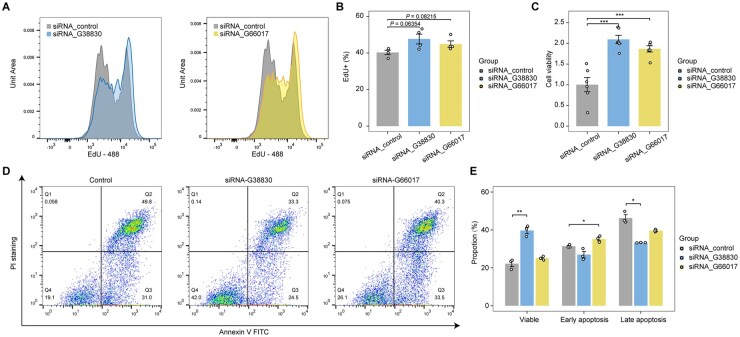
Knockdown of Group 3-specific hervRNAs promotes tumor cell proliferation and attenuates apoptosis. (A) Representative flow cytometry density plots evaluating cell proliferation via EdU assays. The distributions of EdU-488 fluorescence intensity in D283 cells are shown following the siRNA-mediated knockdown of *hervRNA_G38830* (left) and *hervRNA_G66017* (right) compared to the non-targeting control. (B) Quantitative bar chart displaying the percentage of EdU-positive cells derived from the flow cytometry data. Depletion of both hervRNAs results in a marked upward trend in DNA synthesis and cellular proliferation capacity. (C) Assessment of overall cell viability using the Cell Counting Kit-8 (CCK-8) assay in D283 cells. The targeted knockdown of *hervRNA_G38830* and *hervRNA_G66017* leads to a significant increase in cell viability. (D) Representative flow cytometry scatter plots for apoptosis analysis using Annexin V-FITC and PI dual staining. The quadrants demarcate viable cells (Q4: Annexin V−/PI−), early apoptotic cells (Q3: Annexin V+/PI−), and late apoptotic/necrotic cells (Q2: Annexin V+/PI+). (E) Quantitative analysis of the apoptotic cell proportions. The targeted knockdown of *hervRNA_G38830* significantly reduces both early and late apoptotic population. The anti-apoptotic phenotype following *hervRNA_G66017* knockdown is also observed, albeit less pronounced. All bar chart data are presented as mean ± SE. Statistical significance was evaluated using two-sided Student’s *t*-tests. *, *P* < .05. **, *P* < .01. ***, *P* < .001.

Given GSEA-implicated regulation of cell proliferation and apoptosis, we assess cell viability after *hervRNA_G38830* and *hervRNA_G66017* knockdown using EdU fluorescence-activated cell sorting (FACS) assays and Cell Counting Kit-8 (CCK-8) experiments. The results of EdU and CCK-8 assays consistently showed enhanced cell viability after *hervRNA_G38830* and *hervRNA_G66017* knockdown (Figure 6A-C). Furthermore, cell apoptosis assays by flow cytometry with Annexin-V detection revealed a significantly reduced apoptosis rate after *hervRNA_G38830* knockdown, while the apoptosis phenotype of *hervRNA_G66017* knockdown was less pronounced (Figure 6D and E). Collectively, our proof-of-concept experiments suggested that hervRNAs could inhibit cellular proliferation and promote apoptosis in Group 3 MB. Additionally, validation of a SHH-specific candidate, *hervRNA_G14467*, in DAOY cells demonstrated that its knockdown significantly inhibited cell proliferation (Supplementary Figure S7A and B), further highlighting the functional heterogeneity of hervRNAs across different MB subgroups.

### Clinical Application of hervRNA for Medulloblastoma Subgroup Classification

To further evaluate independently expressed hervRNAs for molecular subgroup classification, we applied NMF algorithm and consensus clustering with expression profiles of 4121 independent hervRNAs with TPM > 1 to classified 63 MB samples, which accurately matched 59 samples to consensus subgroups (Supplementary Figure 8A). However, the NMF algorithm relied on precise expression values, and was susceptible to individual heterogeneity and sparse expression pattern of hervRNA. Therefore, we developed a semi-quantitative method based on the binary expression status of hervRNAs. By classifying hervRNA expression as either present or absent, we assessed the discriminatory power of each hervRNA in distinguishing the four subgroups (Supplementary Figure 8B, Supplementary Table S7), resulting in 278 hervRNA classifiers. Given their clustered genomic distribution, these classifiers were grouped into 70 hervRNA clusters. Expression of any hervRNA within a cluster defined a cluster positivity, and the subgroup with the highest cluster positivity was defined as the final subgroup. This semi-quantitative classification method accurately classified 61 of 63 samples. Additionally, 37 of 40 samples and 129 of 136 samples from two publicly independent datasets (EGAD00001001620 and EGAD00001004435) were correctly classified, demonstrating the robustness of this approach (Supplementary Figure 8C). These findings underscored the potential of hervRNA as molecular classification markers.

## Discussion

The intricate and poorly understood pathogenesis of MB hinders molecular targeted therapy development and less toxic treatment options, underscoring the urgent need to uncover novel molecular mechanisms. hervRNAs have been implicated in the development and progression of various cancers,^21^^,^^22^^,^^40^ but their role in MB remains unexplored. In this study, we systematically identified the transcriptional landscape of locus-specific hervRNAs in MB. Our findings demonstrated that expression profile of hervRNAs separated MB samples into the four consensus molecular subgroups distinctly but also share the similarity with hervRNA transcriptome of developing cerebellum at 4-7 pcw, suggesting the involvement of hervRNAs in the tumor biology of MB. Following functional annotation, 145 hervRNA candidates, which were transcribed independently from adjacent protein-coding genes, were found to participated in several pathways consistent with previous studies, such as activation of the Hedgehog signaling pathway in SHH MB,^7^^,^^31^^,^^32^ and phototransduction cascade in Group 3 MB,^9^^,^^34^ supporting the robustness of our findings. Besides subgroup-related pathways, hervRNAs also revealed novel pathways related to MB pathogenesis, including negative regulation of class C GPCR, regulated necrosis and DNA methylation in Group 3, as well as ECM remodeling and negative regulation of Rho GTPases in Group 4. Among these candidates, *hervRNA_G38830* and *hervRNA_G66017,* associated with overall survival in Group 3, were experimentally proved to inhibit tumor cell proliferation and promote apoptosis, offering direct evidence linking specific hervRNAs to critical processes in MB tumorigenesis. Our study provided a foundational understanding of hervRNAs expression pattern in MB and uncovered a new layer of complexity in the molecular mechanism of MB tumorigenesis, which might provide novel molecular targets for less toxic and more targeted therapy.

Functional annotation revealed hervRNAs link novel pathways to MB pathogenesis, especially in Group 3. The pathway of negative DNA methylation regulation potentially explained the abnormal activation of certain hervRNAs due to hypomethylation. Additionally, the enrichment of GPCR signaling suggested that hervRNAs might inhibit tumor migration by modulating G protein-coupled receptor kinases 6, previously shown to suppress CXCR4 signaling and inhibit MB cell migration.^41^ The identification of regulated necrosis and pyroptosis pathways was particularly intriguing, as these processes are associated with programmed cell death and anticancer immune responses,^42^ which warrants further investigation in MB. Furthermore, the pathway enrichment of “response to amino acid deficiency” suggested that hervRNAs might help sustain the metabolic demands of rapidly proliferating MB cells, giving them a growth advantage under nutrient-limited conditions.^43^ Specifically, *hervRNA_G38830* and *hervRNA_G66017* were experimentally validated as tumor suppressors that inhibit MB cell proliferation and promote apoptosis. Our differential expression analysis showed that knockdown of these hervRNAs significantly up-regulated critical early neurodevelopmental transcription factors, including *NeuroD2* and *FOXP4*. Coherently, this transcriptomic alteration converged on the activation of classical developmental programs, such as SHH signaling pathway and epithelial-mesenchymal transition (EMT). Given that the aberrant reactivation of SHH and EMT is a well-documented driver of MB progression,^44^^,^^45^ our findings suggest that *hervRNA_G38830* and *hervRNA_G66017* may act as essential molecular brakes to restrict the excessive reactivation of these primitive embryonic hindbrain programs.

Throughout evolution, most HERVs are repressed by host mechanisms like DNA methylation and histone modification.^46^ Despite some hervRNAs are expressed, their transcription was considered to be passively transcribed because of their proximity to active genes. Consistently, we found that nearly half of hervRNAs, especially intronic hervRNAs, were co-expressed with their nearest protein-coding gene. Almost all these correlated hervRNAs were located within 1 Mbp of the nearest protein-coding gene, revealing the detailed interactive distance of protein-coding gene’s impact on hervRNA transcription. This spatial constraint aligns with the span of topologically associating domains (TADs; typically 200-1,000 kbp),^47^ suggesting TAD-mediated compartmentalization coordinates co-expression between hervRNAs and adjacent genes. Therefore, the 1 Mbp was recommended as a key threshold for functional hervRNA screening pipeline to distinguish independently expressed hervRNAs from those passively transcribed.

Traditional molecular classification of MB is mainly based on immunohistochemistry,^48^ gene expression assays,^49^ and DNA methylation arrays.^50^ However, we found hervRNAs also have the potential for molecular classification. Considering the sparse expression patterns of hervRNAs, we developed a novel classification model based on binary values with accuracy > 94.8%. Given hervRNAs represent a novel molecular class that has largely been uncaptured by conventional mRNA and lncRNA profiling, they could provide a valuable and independent layer of complementary diagnostic information. From a translational perspective, adapting this binary classification framework into multiplex RT-qPCR assays might also provide a more cost-effective complement to expensive genome-wide DNA methylation arrays. Integrated with protein-coding genes and DNA methylation information, hervRNAs may have the potential to complement traditional methods and achieve a more accurate molecular classification in the future. This binary method also provides a practical framework for future studies analyzing hervRNA data. In addition to molecular classification, hervRNAs also show potential for application in early diagnosis. hervRNA expression landscape in MB shares notable similarities to the normal hindbrain at 4-7 pcw. Given the sensitivity of hervRNA expression, hervRNAs might capture the slight change of abnormal expression at an early stage of tumor progression during 4-7 pcw. Besides, hervRNA candidates, such as *hervRNA_G38830* and *hervRNA_G66017*, offer possibilities for RNA-based treatment. By targeting these hervRNAs, new therapies could be developed to selectively silence harmful transcripts by RNA interference or antisense oligonucleotide technologies, which might provide potential precision strategies adapted to different MB subgroups.

While our study provides novel insights into the role of hervRNAs in medulloblastoma, there are several limitations. Our study lack of matched Hi-C sequencing for our RNA-seq cohort, which prevents a definitive validation of the 3D spatial relationships between hervRNAs and their adjacent genes. Future studies incorporating 3D genomic profiling and transcriptomic sequencing are warranted to more precisely delineate the regulatory networks of hervRNAs in medulloblastoma. While knockdown experiments demonstrated the functional relevance of specific hervRNAs, our traditional exogenous overexpression assays did not yield highly significant phenotypes. This is likely due to stoichiometric saturation of their endogenous targets or RNA misfolding caused by vector-derived 5’ and 3’ terminal sequences, underscoring the future need for precise structural-preserving methodologies, such as ribozyme-flanked expression vectors or CRISPR activation, to accurately evaluate their gain-of-function effects. Although we provided functional evidence for specific hervRNAs in Group 3 and SHH cell lines, our study still lack of validation for WNT and Group 4 subgroups due to the limited availability of representative cell models. Future studies utilizing advanced models, such as patient-derived xenografts, will be expected to fully elucidate the functional landscape of hervRNAs across all MB subgroups.

## Supplementary Material

vdag109_Supplementary_Data

## Data Availability

The datasets generated during the current study are available on GSA-human under accession number HRA011170. Other datasets are available from European Genome-phenome Archive (EGAD00001001620, EGAD00001004435, EGAD00001001630) and the European Nucleotide Archive (E-MTAB-6814).
