# Supplementary material for: Systematic decoding the functional role of human endogenous retrovirus-derived RNAs in medulloblastoma
Source: Neurooncol Adv. 2026 Apr 29;8(1):vdag109. doi: 10.1093/noajnl/vdag109 (PMC13215088; doi:10.1093/noajnl/vdag109)
Supplement: vdag109_Supplementary_Data [file vdag109_supplementary_data.zip › Supplementary Table and Figure Legends.docx]

**Figure S1 | Multidimensional annotation of identified hervRNAs in medulloblastoma.**

**(A)** Violin plot comparing the evolutionary conservation of full-length LTRs located within the promoter regions of identified hervRNAs ("Foreground", defined as LTR in hervRNB TSS region of -2000 to +500 bp) against whole-genome LTRs without transcribed hervRNAs ("Background"). Conservation is calculated using the phastCons30way primate metric.

**(B)** Bar chart illustrating the recurrent LTR families in hervRNAs, ranked by the number of linked transcripts and colored by their mean phastCons scores.

**(C)** Lollipop plot showing representative hervRNAs with highly conserved Transcription Factor Binding Sites (TFBS) motif within their LTR promoters. Defined by a strict constraint threshold of phastCons > 0.5. Line length indicates the mean phastCons score of the conserved motifs per hervRNA, dot size represents the absolute number of conserved motifs of the hervRNA, and color mapping reflects the enrichment *P*-value of the corresponding motif shown on the left.

**(D)** Bar chart detailing the open reading frame (ORF) integrity of the identified hervRNAs predicted by TransDecoder.

**(E-F)** Histograms depicting the global folding stability of hervRNAs predicted by RNAfold, evaluated by **(E)** normalized minimum free energy (MFE) and **(F)** normalized ensemble diversity. The dashed lines indicate the thresholds (normalized MFE ≤ -0.25 kcal/mol/nt and diversity ≤ 0.15/nt) applied to identify the 35.98% of hervRNAs capable of forming highly stable secondary structures.

**(G-H)** Bar charts summarizing the integrative functional potential of **(G)** all 39,613 expressed hervRNAs and **(H)** the 9,815 filtered independently expressed hervRNAs. Transcripts are grouped by the number of satisfied criteria (LTR primate-constraint motif, complete ORF, and stable global RNA fold).

**(I)** Heatmap evaluating the functional characteristics of the subgroup-specific hervRNAs across the four MB subgroups. The values denote the percentage of candidates meeting each criterion.

**Figure S2 | NMF-based molecular subgrouping and developmental dynamic expression trajectories of hervRNAs.**

**(A)** Consensus clustering heatmap based on Non-negative Matrix Factorization (NMF) of the 63 MB patient samples using the expression profiles of identified hervRNAs. The clustering delineates a clear stratification into the four consensus molecular subgroups (WNT, SHH, Group 3, and Group 4), as indicated by the top annotation bars and high silhouette scores.

**(B)** Line plot illustrating the overall developmental dynamic expression trajectory (mean TPM) of the 1,365 shared hervRNAs across normal cerebellum development (derived from the E-MTAB-6814 dataset). The x-axis represents developmental time points. The vertical grey dashed line demarcates the precise time of birth, highlighting global transcriptional shifts between fetal and postnatal stages.

**(C)** Line plots detailing the specific expression profiles (TPM) of 12 representative hervRNAs across normal cerebellar development, categorizing them into three distinct temporal patterns: steadily increasing expression post-birth, high expression strictly during prenatal/fetal stages followed by postnatal down-regulatio, and transient peak expression precisely at the time of birth. The vertical grey dashed line indicates the time of birth.

**Figure S3 | The characteristics of independently expressed hervRNAs in MB**

**(A)** Line plot displaying the parameter sensitivity analysis used to define the genomic distance boundary for hervRNA-gene co-expression. The y-axis represents the inclusion ratio of highly correlated nearest-neighbor pairs across increasing distance thresholds (x-axis). The curve exhibits an unconstrained knee at 0.7 Mbp and reaches a near-saturation plateau (>0.999) at 1.1 Mbp.

**(B)** Density plot illustrating the distribution of Topologically Associating Domain (TAD) sizes across the four consensus MB molecular subgroups. Based on re-analyzed Hi-C sequencing data (dataset GSE246125), the average TAD size is approximately 1.5 Mbp across all subgroups (WNT: ~1.58 Mbp; SHH: ~1.60 Mbp; Group 3: ~1.46 Mbp; Group 4: ~1.51 Mbp).

**(C)** Violin plot comparing the expression levels (log_2_(TPM+1)) between passively co-expressed and independently expressed hervRNAs, stratified by intergenic status.

**(D)** Heatmaps displaying the hierarchical clustering of functional pathways for subgroup-specific hervRNAs across the four MB subgroups. Functional classes were defined based on Normalized Enrichment Scores (NES) derived from Gene Set Enrichment Analysis (GSEA) of hervRNA-co-expressed protein-coding genes using the Reactome pathway database.

**Figure S4 | Module-trait relationships defining subgroup-specific co-expression networks.**

**(A-D)** Heatmaps illustrating the correlation between specific WGCNA module eigengenes (y-axis) and the binary clinical traits representing the four consensus medulloblastoma molecular subgroups (x-axis; e.g., "WNT or not"). The panels display the correlation matrices for the differentially expressed hervRNA (DEH) modules containing **(A)** WNT-specific, **(B)** SHH-specific, **(C)** Group 3-specific, and **(D)** Group 4-specific hervRNAs. The numerical values within each cell represent the Pearson correlation coefficients. The color gradient denotes the strength and direction of the association.

**Figure S5 | Independent cross-cohort validation of the subgroup-specific expression patterns of hervRNAs.**

**(A)** Heatmap illustrating the absolute expression levels (Transcript Per Million, TPM) of the 13 differentially hypomethylated, Group 3-specific hervRNAs within an external validation RNA-seq cohort (EGAD00001001620, matched to the methylation dataset).

**(B)** Comprehensive heatmap depicting the expression profiles of the identified subgroup-specific independently expressed hervRNAs across a separate, large-scale independent clinical RNA-seq cohort (dataset EGAD00001004435). Expression values (TPM) are Z-score scaled by row to highlight relative inter-sample differences.

**Figure S6 | Gene Set Enrichment Analysis (GSEA) revealing the suppression of key tumor-suppressive and immune pathways following hervRNA knockdown.**

**(A)** GSEA enrichment plot demonstrating the significant down-regulation of the P53 signaling network (HALLMARK_P53_PATHWAY) following the siRNA-mediated knockdown of *hervRNA_G38830*. The upper panel displays the distribution of individual pathway-associated genes (vertical yellow bars) across the pre-ranked list of differentially expressed genes. The lower panel exhibits the Running Enrichment Score (RES, blue continuous line).

**(B)** GSEA enrichment plot illustrating the significant down-regulation of the class I MHC peptide loading pathway following the knockdown of *hervRNA_G66017*.

**Figure S7 | Experimental validation of the SHH-specific *hervRNA_G14467* in DAOY cells.**

**(A)** Representative agarose gel electrophoresis image confirming the endogenous transcription of the SHH-specific candidate *hervRNA_G14467* in the SHH MB cell line DAOY via reverse transcription PCR (RT-PCR). The left lane displays the standard DNA marker ladder for size reference.

**(B)** Cell Counting Kit-8 (CCK-8) assay evaluating the proliferation capacity of DAOY cells over a 4-day period following siRNA-mediated knockdown of *hervRNA_G14467* compared to a non-targeting negative control (NC) by *t*-test. Absorbance was measured at 450 nm. Data are presented as mean ± SE . *, *P* < 0.05. **, *P* < 0.01.

**Figure S8 | Development and independent validation of semi-quantitative hervRNA-based molecular classification model.**

**(A)** Consensus clustering heatmap based on the Non-negative Matrix Factorization (NMF) algorithm, utilizing the continuous expression profiles of 4,121 independently expressed hervRNAs across 63 MB samples.

**(B)** Scatter plots evaluating the diagnostic discriminatory power of individual hervRNAs utilizing a transformed binary expression framework (presence versus absence) to overcome expression sparsity. Panels represent the four molecular subgroups (Top left: WNT; top right: SHH; bottom left: Group 3; bottom right: Group 4). The axes denote Specificity and Sensitivity, with each dot representing a candidate hervRNA classifier colored by its statistical significance.

**(C)** Receiver Operating Characteristic (ROC) curves demonstrating the highly accurate diagnostic performance of the final 70-cluster semi-quantitative classification model across the four MB subgroups. The model's robustness and broad generalizability are validated across three independent clinical cohorts: Dataset 1 (our in-house discovery cohort, accurately classifying 61 of 63 samples), Dataset 2 (public cohort EGAD00001004435, correctly classifying 129 of 136 samples), and Dataset 3 (public cohort EGAD00001001620, correctly classifying 37 of 40 samples)

**Table S1** | Sample metadata

**Table S2** | The characteristics of hervRNAs identified in MB samples

**Table S3** | Multidimensional annotation of identified hervRNAs in medulloblastoma

**Table S4** | Differential expression analysis of subgroup-specific independently expressed hervRNAs

**Table S5** | The top 5 enriched terms ranked by |NES| for each subgroup-specific hervRNA

**Table S6** | Differentially expressed gene of G38830 knockdown and G66017 knockdown

**Table S7** | Evaluation of classification based on hervRNA

**Table S8** | Primers design

**Table S9** | siRNA design
