# Supplementary figures and images for: Systematic decoding the functional role of human endogenous retrovirus-derived RNAs in medulloblastoma
Source: Neurooncol Adv. 2026 Apr 29;8(1):vdag109. doi: 10.1093/noajnl/vdag109 (PMC13215088; doi:10.1093/noajnl/vdag109)

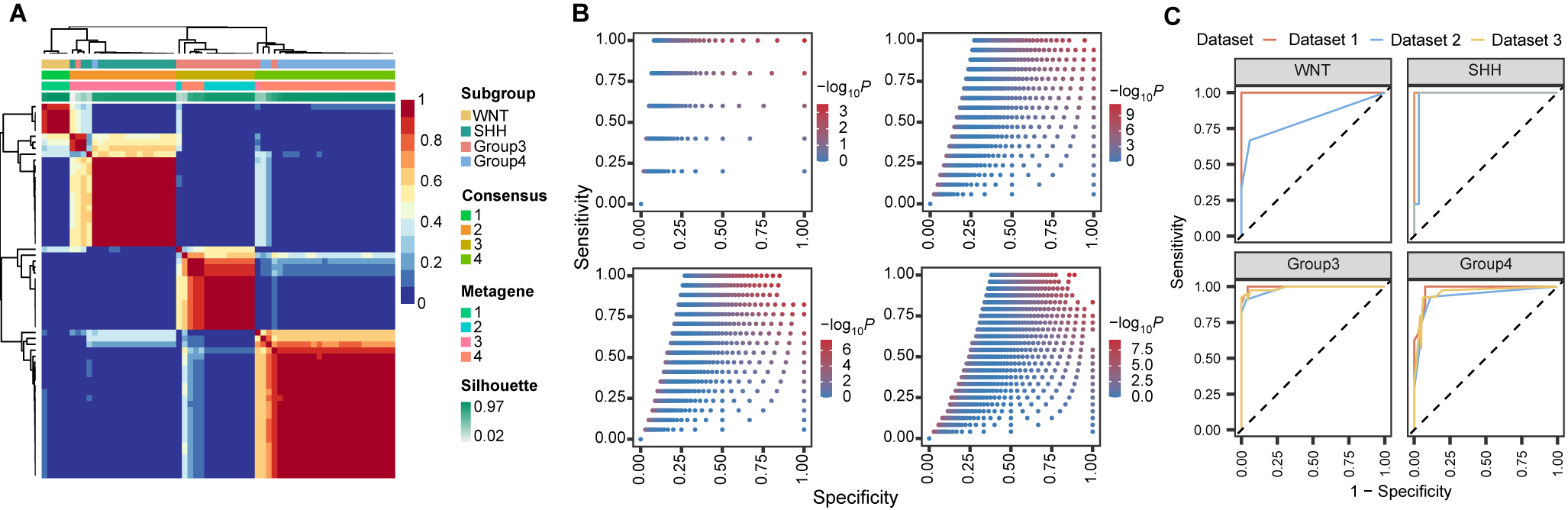

Supplement: vdag109_Supplementary_Data [file vdag109_supplementary_data.zip › FigureS8.tif]

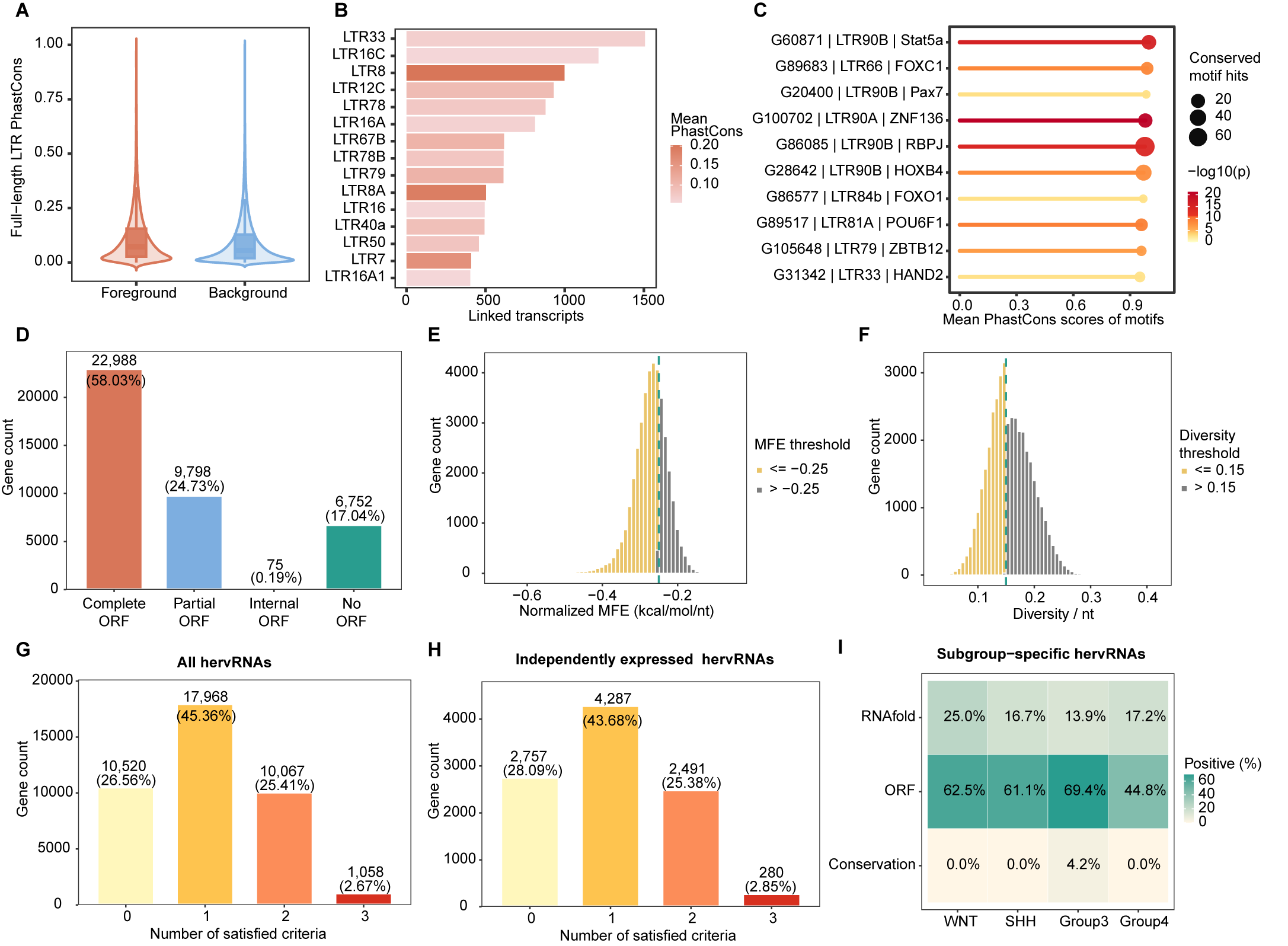

Supplement: vdag109_Supplementary_Data [file vdag109_supplementary_data.zip › FigureS1.tif]

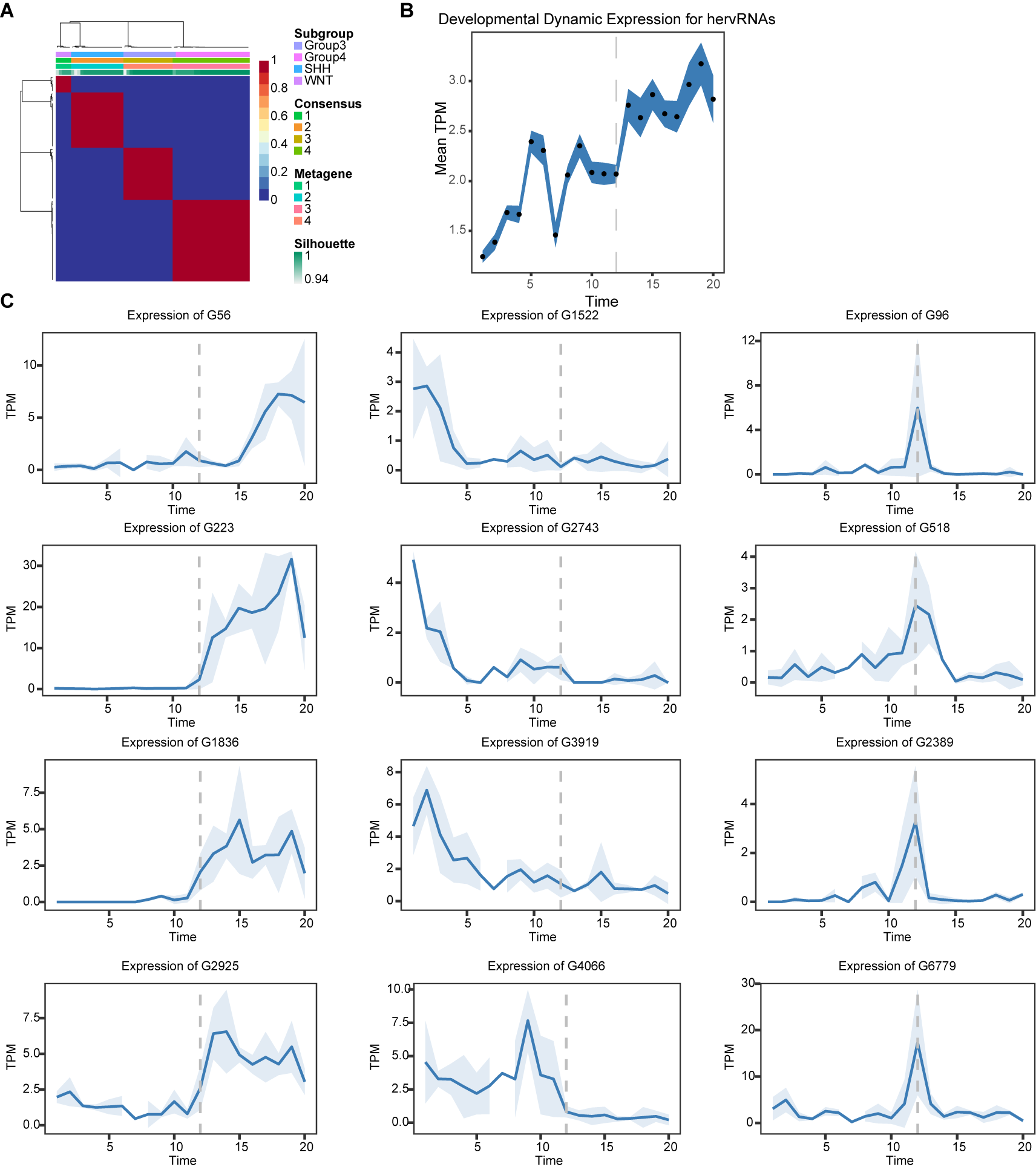

Supplement: vdag109_Supplementary_Data [file vdag109_supplementary_data.zip › FigureS2.tif]

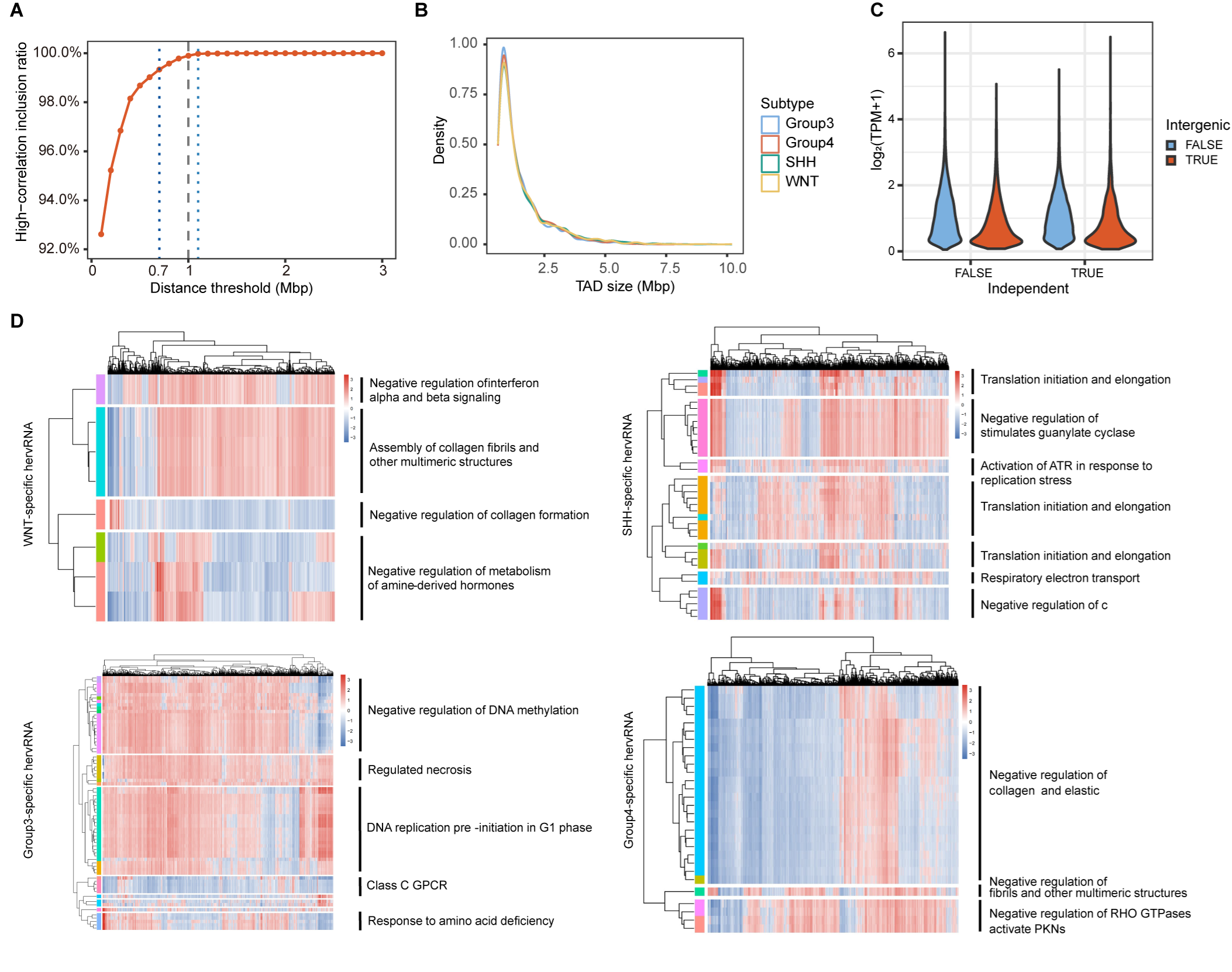

Supplement: vdag109_Supplementary_Data [file vdag109_supplementary_data.zip › FigureS3.tif]

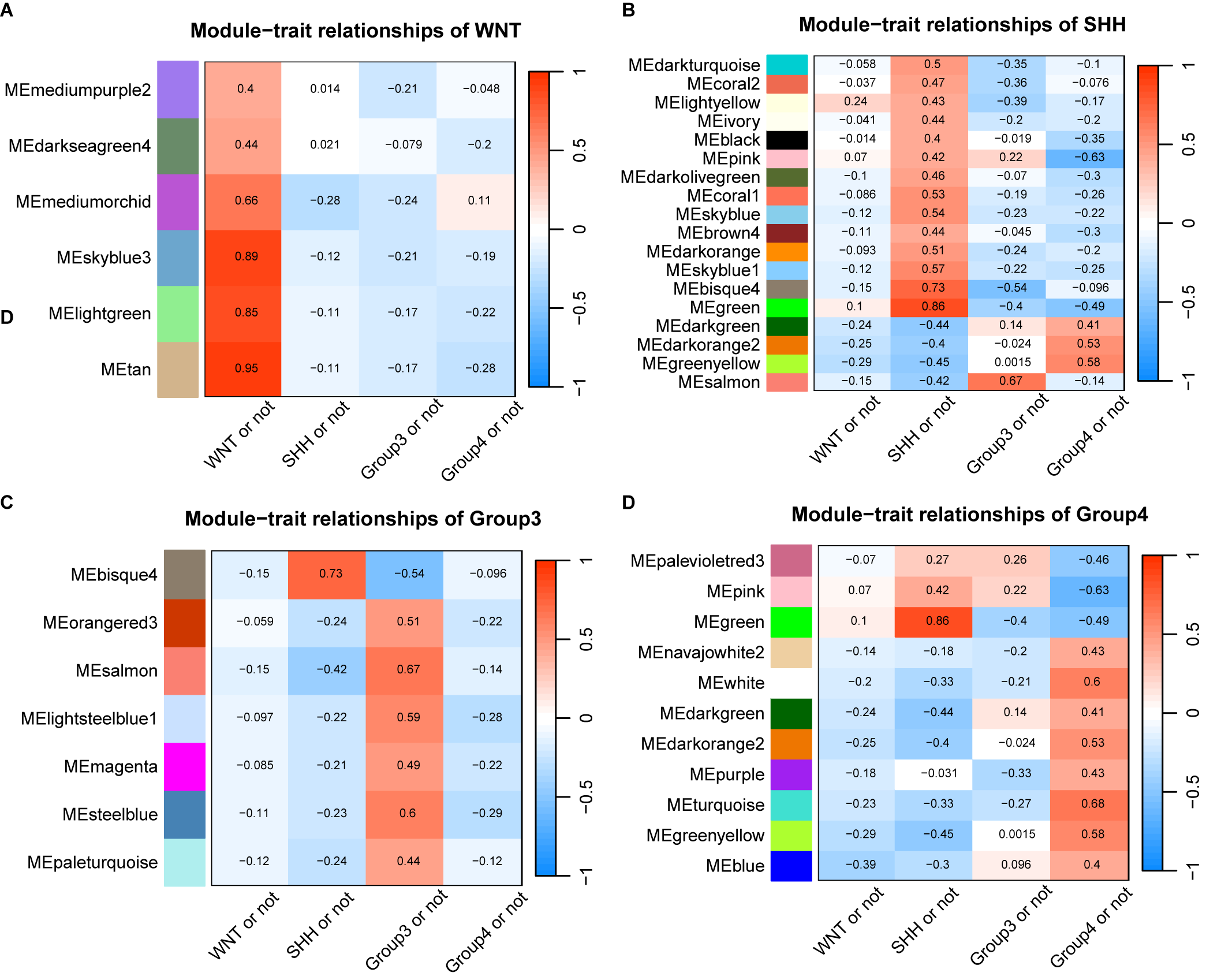

Supplement: vdag109_Supplementary_Data [file vdag109_supplementary_data.zip › FigureS4.tif]

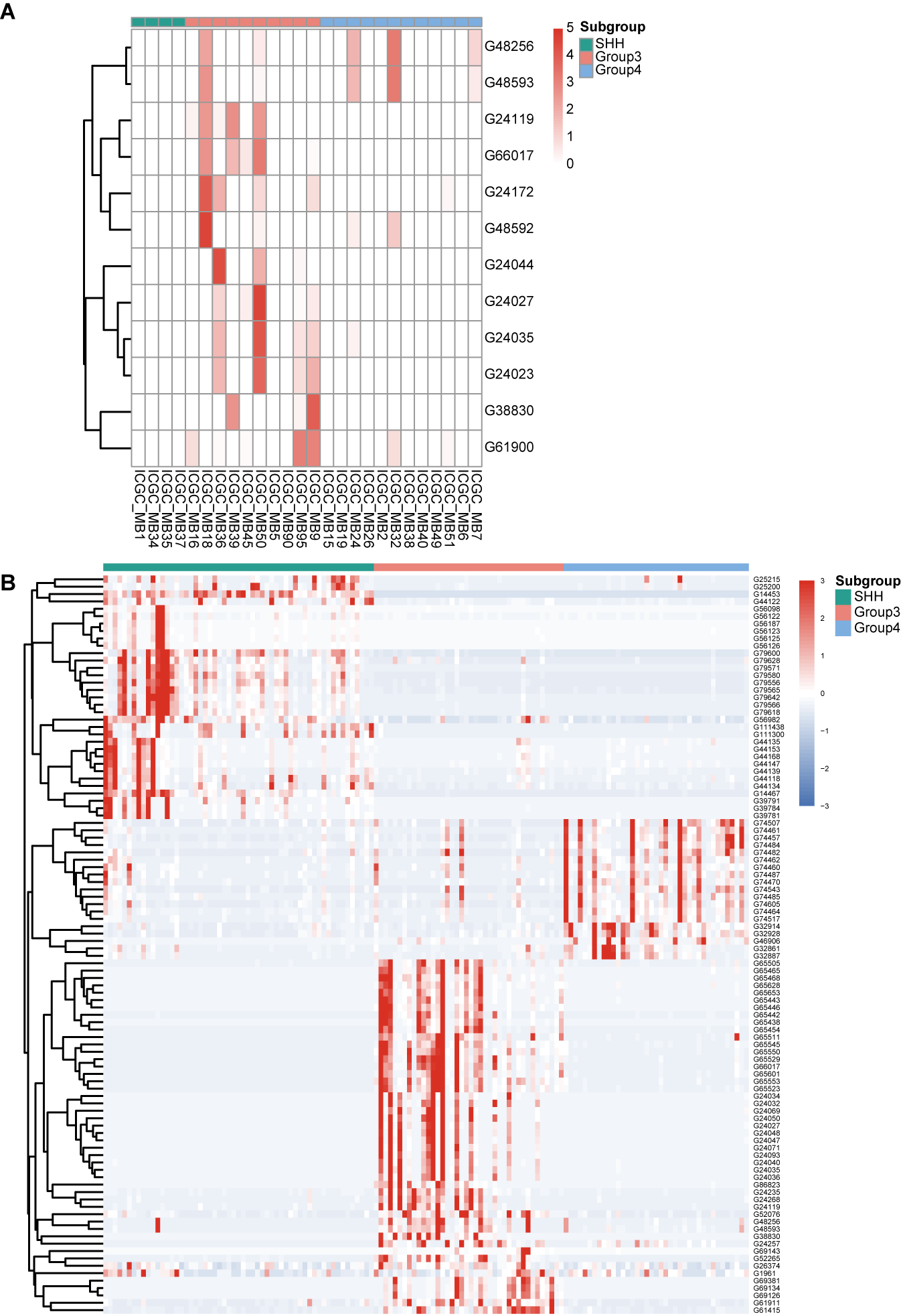

Supplement: vdag109_Supplementary_Data [file vdag109_supplementary_data.zip › FigureS5.tif]

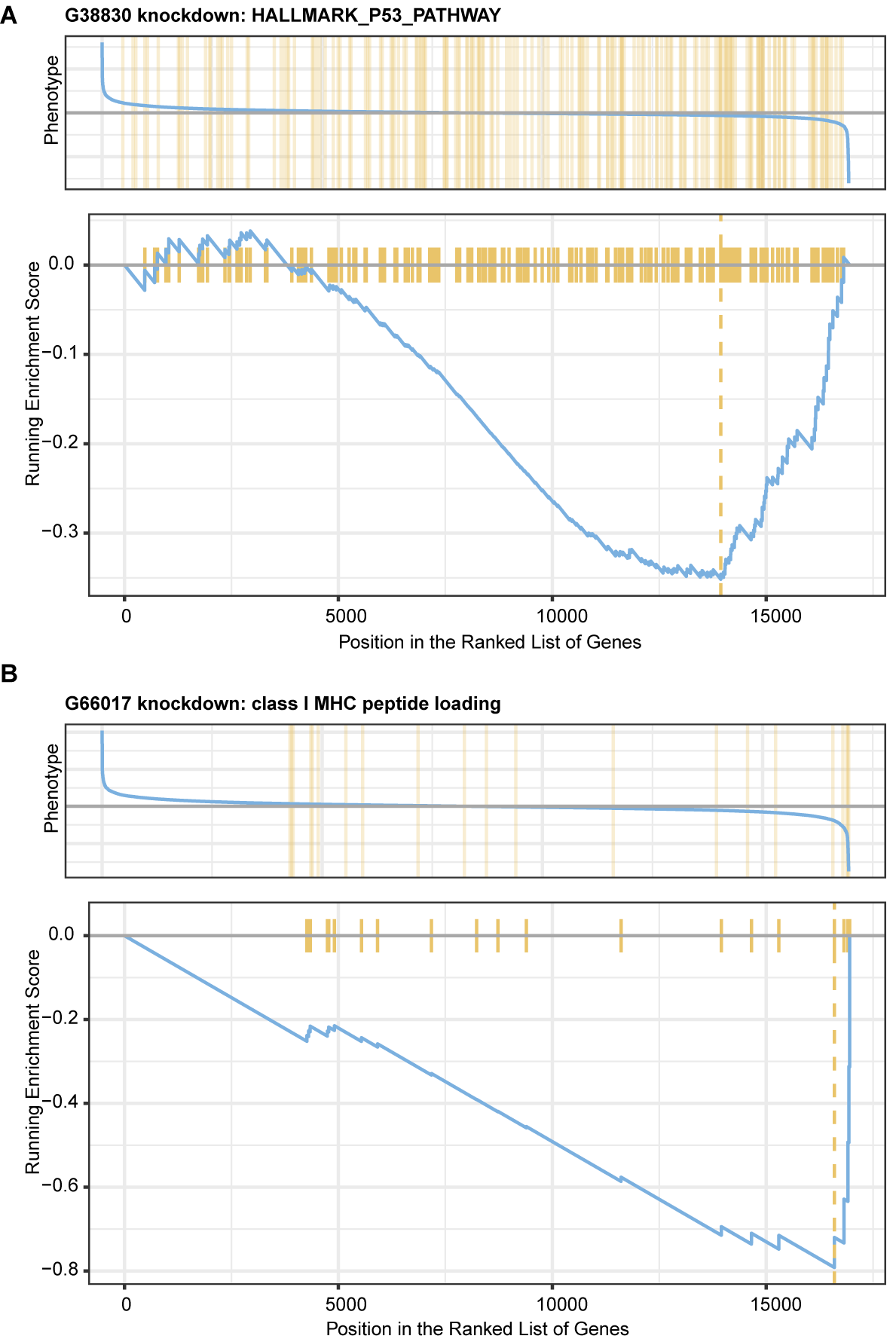

Supplement: vdag109_Supplementary_Data [file vdag109_supplementary_data.zip › FigureS6.tif]

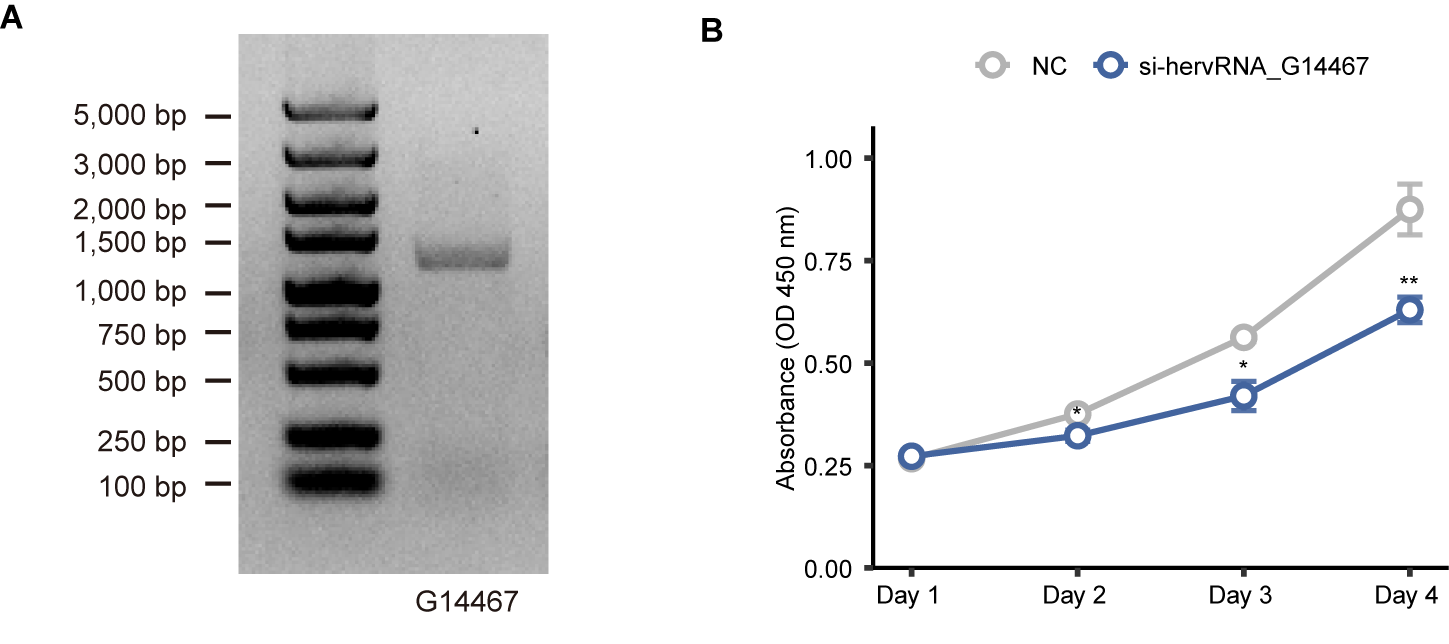

Supplement: vdag109_Supplementary_Data [file vdag109_supplementary_data.zip › FigureS7.tif]
